# Three-Dimensional Culture Decreases the Angiogenic Ability of Mouse Macrophages

**DOI:** 10.3389/fimmu.2021.795066

**Published:** 2021-12-22

**Authors:** Haoxin Shi, Dong Li, Qing Shi, Zhenxia Han, Yuwei Tan, Xiaodong Mu, Miao Qin, Zengjun Li

**Affiliations:** ^1^ Endoscopy Room, Department of Gastroenterology, Shandong Cancer Hospital and Institute, Shandong First Medical University and Shandong Academy of Medical Science, Jinan, China; ^2^ Cryomedicine Lab of Qilu Hospital, Shandong University, Jinan, China; ^3^ Department of Geriatric Medicine, Qilu Hospital of Shandong University, Jinan, China; ^4^ College of Pharmacy and Pharmaceutical Sciences, Shandong First Medical University (Shandong Academy of Medical Sciences), Jinan, China

**Keywords:** 3D culture, macrophage, angiogenesis, collagen microcarrier, VEGFA, ANG2

## Abstract

Macrophages play important roles in angiogenesis; however, previous studies on macrophage angiogenesis have focused on traditional 2D cultures. In this study, we established a 3D culture system for macrophages using collagen microcarriers and assessed the effect of 3D culture on their angiogenic capabilities. Macrophages grown in 3D culture displayed a significantly different morphology and arrangement under electron microscopy compared to those grown in 2D culture. Tube formation assays and chick embryo chorioallantoic membrane assays further revealed that 3D-cultured macrophages were less angiogenic than those in 2D culture. Whole-transcriptome sequencing showed that nearly 40% of genes were significantly differently expressed, including nine important angiogenic factors of which seven had been downregulated. In addition, the expression of almost all genes related to two important angiogenic pathways was decreased in 3D-cultured macrophages, including the two key angiogenic factors, VEGFA and ANG2. Together, the findings of our study improve our understanding of angiogenesis and 3D macrophage culture in tissues, and provide new avenues and methods for future research on macrophages.

## Introduction

Macrophages are a type of immune cell that are widely distributed in all bodily tissues and play vital roles in development, homeostasis, tissue repair, and immunity (1). Consequently, macrophages have long been a popular research topic for scientists. Macrophages are functionally heterogeneous cells that are susceptible to changes in their microenvironment and are shaped by various stimuli. Activated macrophages can be divided into two classes: classically polarized macrophages (M1), which are induced by interferon (IFN)-γ, lipopolysaccharide (LPS), and interleukin (IL)-1β; and alternatively activated macrophages (M2), which are activated by IL-4 and IL-13 (2, 3). While M1 macrophages can promote inflammatory responses, M2 macrophages exert anti-inflammatory functions in wound healing and tumor progression (4). Macrophages also play a vital role in promoting angiogenesis by secreting various pro-angiogenetic cytokines and growth factors, such as angiopoietin-2 (Ang2), basic fibroblast growth factor (bFGF, FGF2), vascular endothelial growth factor A (VEGFA), and matrix metalloproteinase (MMP)-2. Consequently, it is important to study macrophage angiogenesis for applications in rheumatoid arthritis, wound repair, and glioblastoma.

Traditional macrophage research has mostly been based on two-dimensional (2D) culture, in which cells grow in a monolayer under adherent conditions. However, this does not accurately simulate the environment of the cells in 3D structures *in vivo*, including cell-cell and cell-extracellular matrix (ECM) interactions (5). In 2D culture, cells grow on a flat substrate and thus have a flattened shape with a remodeled internal cytoskeleton (6), which has been shown to alter cellular gene expression (7). These flaws have been confirmed by various previous studies. For example, immortalized tumor cell lines grown in 2D culture systems resulted in a 95% failure rate for drug responses in human subjects, suggesting that the two-dimensional cell culture model may be inadequate for drug development (8). Recently, 3D culture methods have been developed that have significantly improved cell culture by providing the cell with a microenvironment that more closely represents *in vivo* conditions. Compared to cells grown in a 2D environment, those in 3D culture have no polarity, discrete matrix fibers, and variable stiffness. Moreover, these cells adhere in three dimensions and their diffusion and migration are impeded (9–11). The enhanced contact between cells increases intercellular signaling, facilitates developmental processes, and allows cells to differentiate into more complex structures than through traditional culture methods (4, 12).

Although multiple studies have demonstrated the superiority of 3D culture, little is known about the precise effect of 3D culture techniques on macrophage angiogenesis. In this study, we aimed to assess the changes in cell plasticity and function caused by 3D macrophage culture and their effects on angiogenic function. In addition, we evaluated the potential of 3D macrophage culture for biological experiments. First, we used a scaffold to establish a 3D cell culture system and then detected its effect on various cellular characteristics and gene expression. Finally, we demonstrated changes caused by 3D cell culture on pro-angiogenic ability *in vitro* and *in vivo*.

## Materials and Methods

### Cell Acquisition and Culture

RAW264.7 mouse macrophages were purchased from Shanghai Zhong Qiao Xin Zhou Biotechnology Co. Ltd (Beijing, China). The cells were cultured in high-glucose Dulbecco’s modified Eagle’s medium (DMEM; Gibco, Beijing, China) supplemented with 10% fetal bovine serum (FBS; Gibco) and 1% penicillin-streptomycin (Gibco) in a 5% CO_2_ atmosphere incubator at 37°C.

Human umbilical vein endothelial cells (HUVECs) were isolated from normal human umbilical veins were donated by the Cryogenics Laboratory of Qilu Hospital (Jinan, China). Briefly, normal human umbilical cords of approximately 10 cm were placed in a petri dish and two syringes were inserted into the vessel. The vessel was then rinsed with warm phosphate buffered saline (PBS) to remove any blood and injected with 6 mL of 0.25% (w/v) collagenase II and 0.25% (W/V) collagenase IV (Worthington, Biochemical Corp., Lakewood, NJ, USA). After incubation in a humidified 5% CO_2_ atmosphere at 37°C for 60 min, the solution was centrifuged to obtain HUVECs. The cells were cultured in complete endothelial cell medium (#1001; Sciencell, San Diego, CA, USA) containing 10% FBS and endothelial cell growth supplement, and were passaged every three days. The research team that donated the HUVECs identified the cells using CD31 immunofluorescence staining and flow cytometry analysis (13, 14).

### 3D and 2D Culture of RAW264.7 Cells

To establish the 3D cell culture system, cells were seeded on a 3D cell culture scaffold (diameter: 48 wells; TANTTI Laboratory Inc., Taiwan, China). The RAW264.7 cells were suspended in high glucose DMEM at a concentration of 5 × 10^6^/mL and then 80 μL of the suspension was dropped gently onto the scaffold. After immobilization for 30 min in the incubator, 1 mL of complete medium was added to each well and incubation was continued. To establish the 2D culture system, the cells were seeded in a culture bottle under identical conditions as for 3D culture. In both culture methods, the cells were passaged every three days.

### RNA-Seq and Analysis

Total RNA was extracted from purified and untreated RAW264.7 cells from 3D and 2D culture systems using TRIzol (Invitrogen Life Technologies; Carlsbad, CA, USA) according to the manufacturer’s protocols. The RNA samples were analyzed using Whole Genome Oligo Microarrays (one−color; Agilent Technologies, Shanghai, China). After RNA had been hybridized to the microarray, it was washed and scanned, and data were extracted using Agilent Feature Extraction Software (v.11.0.1.1; Agilent Technologies). Gene expression data were generated using Affymetrix GeneChip Human Genome U133 Plus 2.0 on an Affymetrix 3000 instrument (fluidics station and scanner; Thermo Fisher Scientific) running Gene−Chip operating software (v.11.0; Gene Spring Software; Agilent Technologies). DEGs were screened using Degseq, with |log2 fold change| ≥ 1 and *q* value (*p*adj, *p* value after correction) < 0.05 as criteria. Gene Ontology (GO; http://geneontology.org/) and Kyoto Encyclopedia of Genes and Genomes (KEGG; www.genome.jp/kegg/pathway.html) pathway enrichment analyses, were used to identify significantly enriched pathways associated with hematopoietic cell proliferation in DEGs with a fold change ≥ 2.

### Cell Cycle Analysis

After 24 h, 2D- and 3D-cultured RAW264.7 cells were collected, washed twice with PBS, and resuspended in 500 mL PBS. The cell suspension was gently dropped into a tube containing 5 mL 75% ethanol (precooled to -20°C) which was shaken at -20°C for 1 h to fix the cells. Next, 5 × 10^6^ cells per tube were collected, washed twice with PBS (with 1% FBS), resuspended in 500 μL PBS, and treated with RNace (Bestbio, Shanghai, China) for 30 min at 37°C in a water bath. The cells were then collected and 500 μL propidium iodide (PI; Becton, Dickinson, and Company, USA) was added to label the cells. After 15 min in the dark, following the manufacturer’s instructions, the cells were analyzed by flow cytometry (Guava easyCyte8HT; EMD Millipore).

### Scanning Electron Microscope (SEM) Observation

After 24 h, 2D- and 3D-cultured RAW264.7 cells were immobilized in a mixture of 2.5% glutaraldehyde and 4% paraformaldehyde for 24 h, soaked in 0.1 mL phosphoric acid buffer, and fixed in 1% osmium tetroxide for 2 h. The cells were then soaked in double-steamed water, dehydrated using an ethanol gradient, dried in tert-butyl alcohol vacuum, and sputtered with ions. Finally, the results were observed and recorded using thermal field emission SEM (Sigma 300, ZEISS, Germany).

### RNA Extraction and Reverse−Transcription Quantitative PCR (RT−qPCR)

After overnight 2D and 3D culture, total cellular RNA was isolated from RAW264.7 cells using TRIzol^®^ (Invitrogen; Thermo Fisher Scientific) according to the manufacturer’s instructions. First-strand cDNA was synthesized using 1 μg of total RNA with 20 μL of reverse transcriptase reaction mixture using a ReverTraAce qPCR RT Master Mix kit (TOYOBO, Osaka, Japan) with specific primers. RT-qPCR was performed using a Real Time Thermocycler (Analytik Jena AG, qTOWER3G, Germany), and detection was performed with SYBR Green Realtime PCR Master Mix (TOYOBO) in a 20 μL reaction mixture to detect cytokine mRNA levels. The primer sequences used for RT-qPCR are listed in **Supplementary Table 1**. The thermal cycling program was as follows: 95°C for 5 s, followed by 40 cycles of 95°C for 5 s, 53°C for 10 s, and 72°C for 15 s. Data were analyzed using Sequence Detection Software 1.4 (Applied Biosystems, CA, USA). mRNA levels were normalized to the normal group, which was set to 1.

### Gamma-Ray Cell Treatment

To ensure that the cells used for enzyme-linked immunosorbent assay (ELISA) were no longer proliferating and to more accurately measure their ability to secrete factors, we used a medical electron linear accelerator (Varian, clinic 23EX) to deliver radiation (Department of Radiology, Qilu Hospital, Jinan, China). RAW264.7 cells were exposed to 1, 3, 6, and 9 doses of radiation (dose rate: 400 cGy/min). The optimal radiation dose was determined by counting the number of cells before and after 24 h.

### ELISA Detection of ANG-2 and VEGF-A

RAW264.7 cells were seeded at the same concentration (3 × 10^5^ cells/mL) in both 3D and 2D culture systems. To prevent cell proliferation, the cells were treated with γ-RAY at 3 Gy to block cell proliferation. After 24 h, the culture medium from both systems was collected and the cells were counted to show that cell proliferation had been blocked successfully. The supernatant medium was collected for ELISA with a mouse VEGF ELISA kit (EK283/2-01, Multiscience (LIANKE) Biotech, Shanghai, China) and a mouse angiopoietin-2 immunoassay (MANG20, R&D Systems Inc., USA) according to the manufacturer’s instructions. The plates were read using a microplate reader at 450 nm, and the wavelength was corrected to 570 nm. All tests were performed in duplicate.

### Matrigel HUVEC Tube-Formation Assay

Endothelial tube formation assays were performed to measure angiogenesis *in vitro*. To analyze the effect of 3D-cultured macrophages on HUVEC tube formation, we first co-cultured HUVECs with 3D- or 2D-cultured macrophages under the same conditions for 24 h. HUVECs (4 × 10^4^ cells) were placed in the lower layer of the co-culture plate with 500 μL basic medium, while 4 × 10^5^ macrophages were placed in the upper layer with 200 μL DMEM. The 96-well plates and 1 mL tips were precooled for 6 h at -20°C, and the matrix gel (40 μL/well; Corning, NY, USA) was thawed in a refrigerator for 1 d before use. After 12 h, HUVEC branches were observed and imaged under a light contrast microscope. Tube formation was evaluated using Image J (v.1.8.0; National Institutes of Health, Bethesda, MD, USA).

### Chick Embryo Chorioallantoic Membrane (CAM) Assay

Six-day-old chicken embryos were obtained from the Shandong Experimental Breeder Farm (Jinan, China) of No Specific Pathogenic Chickens and incubated at 37°C and 60% humidity. Macrophages cultured in 3D and 2D were suspended in complete medium (10^5^ cells/mL), solidified using 2.4% agar, and divided into small, equal pieces. On the third day, a window of approximately 1 cm in diameter was opened in the shell to detach the CAM from the shell itself, and the underlying CAM vessels were disclosed. Agar plates containing the cells were placed on the CAM separately, and the openings were sealed and placed in an incubator. The chicken embryos were collected in batches from the fifth day to the seventh day and fixed with formaldehyde. The CAM was separated, photographed (the same area at the same focal length), and analyzed using Image J v.1.8.0 (National Institutes of Health).

### Statistical Analysis

GraphPad Prism 7 (GraphPad Software, Inc., La Jolla, CA, USA) was used for statistical analysis. All data were presented as the mean ± SE of three independent experiments. Significant differences between multiple groups were assessed using one-way analysis of variance (ANOVA) with Bonferroni’s test. Statistical analyses were also performed using SPSS Statistics 21.0 (SPSS, Inc., Chicago, IL, USA). A two-sided probability (*p)* of less than 0.05 was regarded as statistically significant.

## Results

### 3D- and 2D-Cultured Macrophages Display Different Morphologies

**Figure 1:**
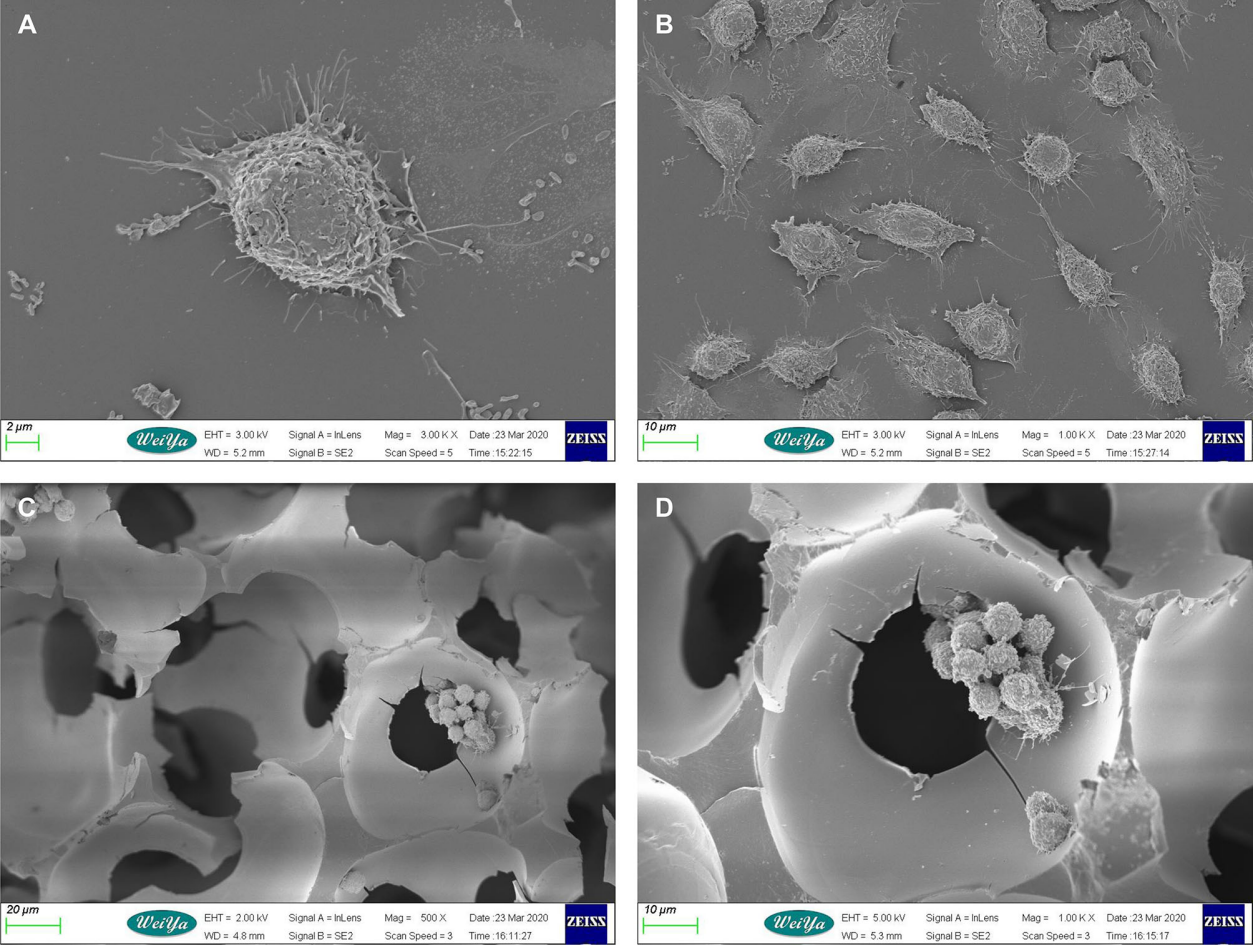
Scanning electron microscopy images of mouse macrophages cultured in 2D and 3D. **(A)** Macrophages in 2D culture. Scale bar: 2 μm. **(B)** Macrophages in 2D culture. Scale bar: 10 μm. **(C)** Macrophages in 3D culture. Scale bar: 10 μm. **(D)** Macrophages in 3D culture. Scale bar: 20 μm.

In order to explore the effect of 3D culture on the morphology of macrophages, we used scanning electron microscopy to observe two groups of macrophages in 3D and 2D culture. The biological morphology and arrangement of macrophages growing on the 3D scaffold differed completely from those growing in 2D (**Figure 1**). Cells on the 3D scaffold grew in clusters and clung to the cavity of the scaffold microenvironment, with the majority of cells being nearly round. Conversely, macrophages cultured in 2D were dispersed and tiled on the plane, and varied in shape from spindle-shaped or round to polygonal.

### More 3D-Cultured Macrophages Were Blocked in the G0/G1 Phase

**Figure 2:**
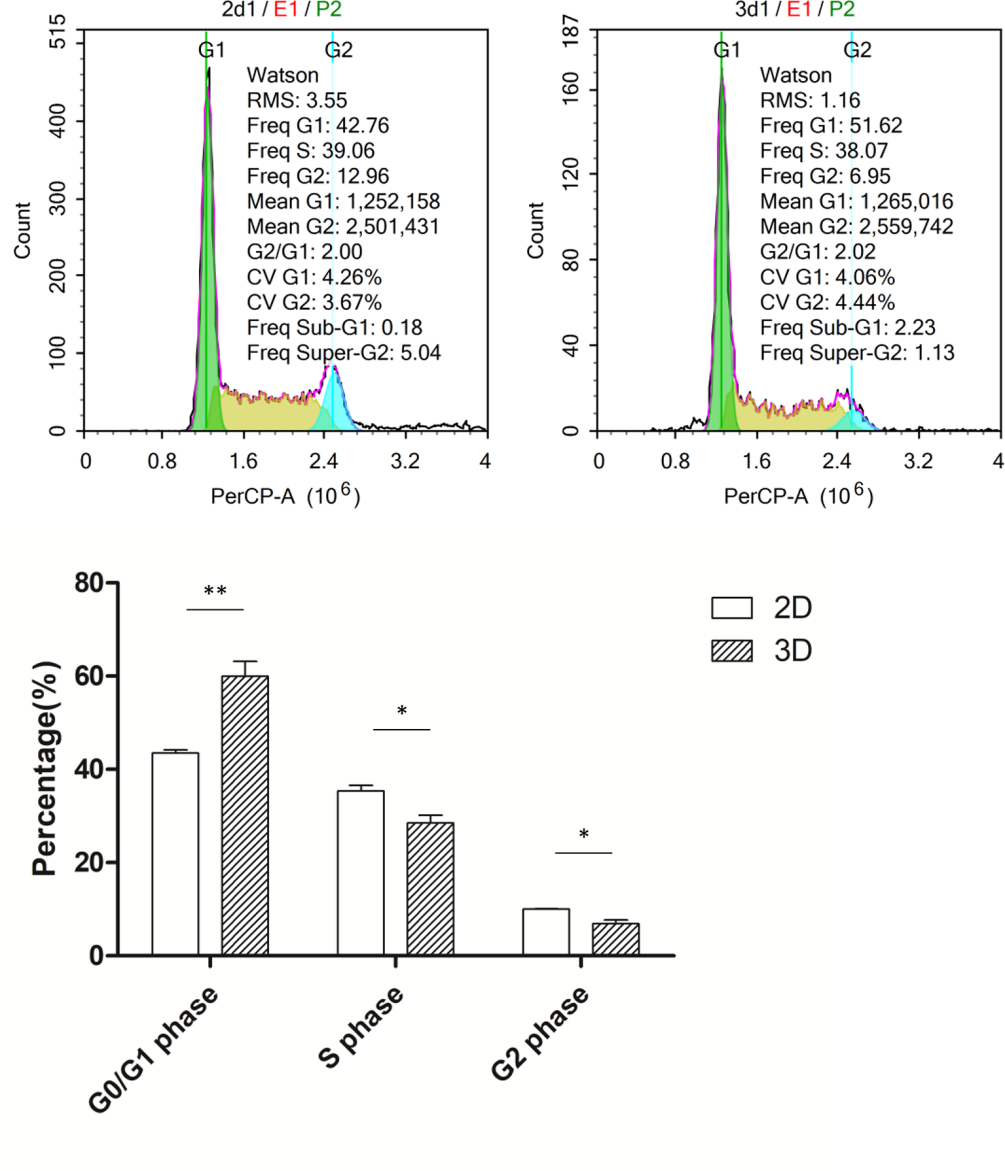
Cell cycle of macrophages in 2D and 3D culture. Error bars represent the SEM (*n* = 3, mean ± SEM; ^*^ *p* < 0.05, ^**^ *p* < 0.01, Student’s *t*-test).

In the preliminary experiment, we found that the growth rate of cells under 3D culture was slower; thus, we first detected the cell cycle of the macrophages. Flow cytometry analysis revealed that cells cultured in 3D had a significantly higher G0/G1 ratio, a lower proportion of cells in G2/M phase, and a slight increase in the proportion of cells in S phase compared to those cultured in 2D (**Figure 2**).

### 3D-Cultured Macrophages Exhibit Reduced Pro-Angiogenic Abilities

**Figure 3:**
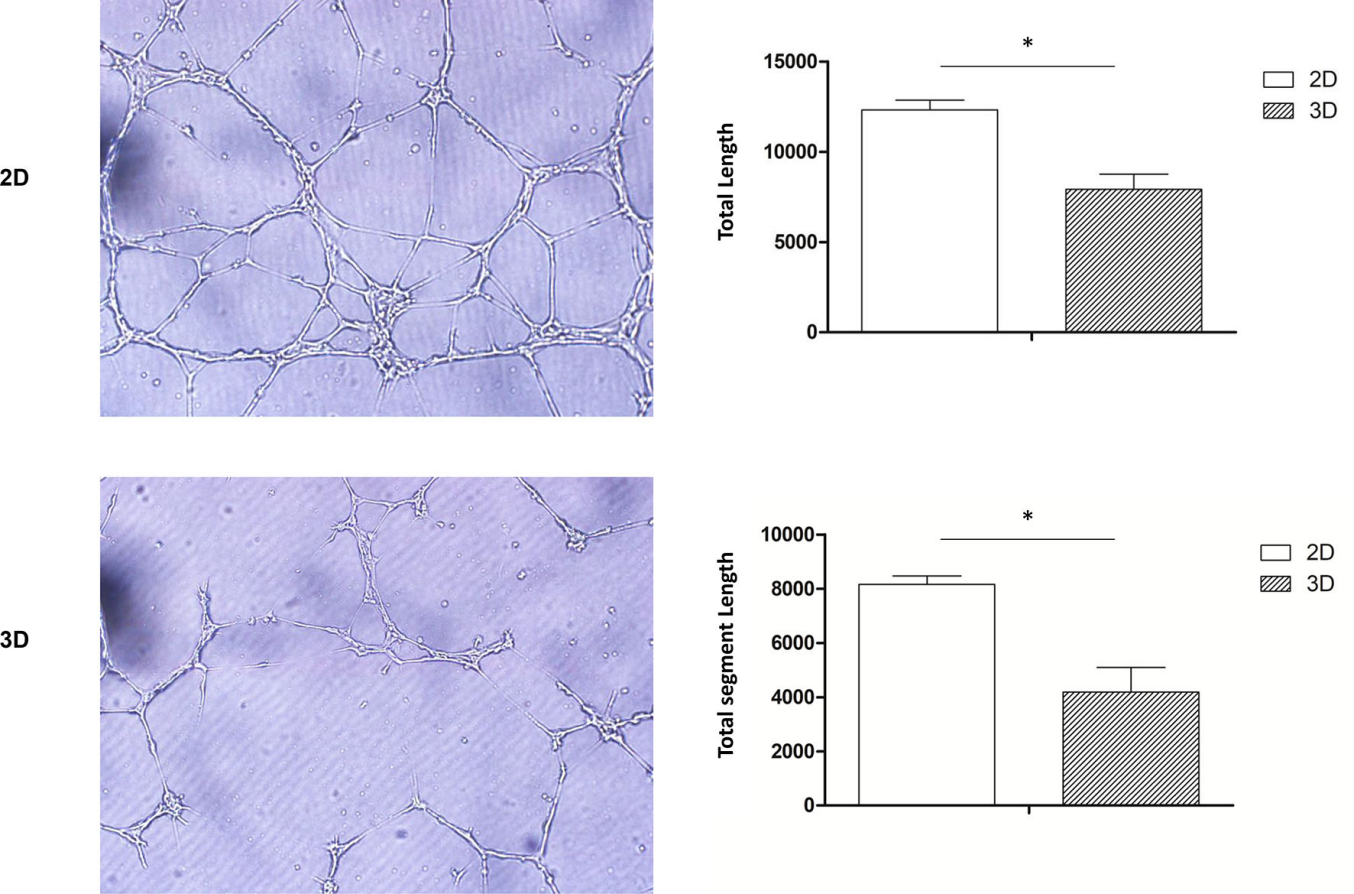
Tube formation assay. HUVECs co-cultured with 2D- and 3D-cultured RAW264.7 cells were observed under a microscope (scale bar: 100 μm) (Olympus, BX53, Japan) and imaged (CellSens, v.1.18, Japan). Data were quantified using imageJ software. Error bars represent the SEM (*n* = 3, mean ± SEM; *p < 0.05, Student’s *t*-test).

**Figure 4:**
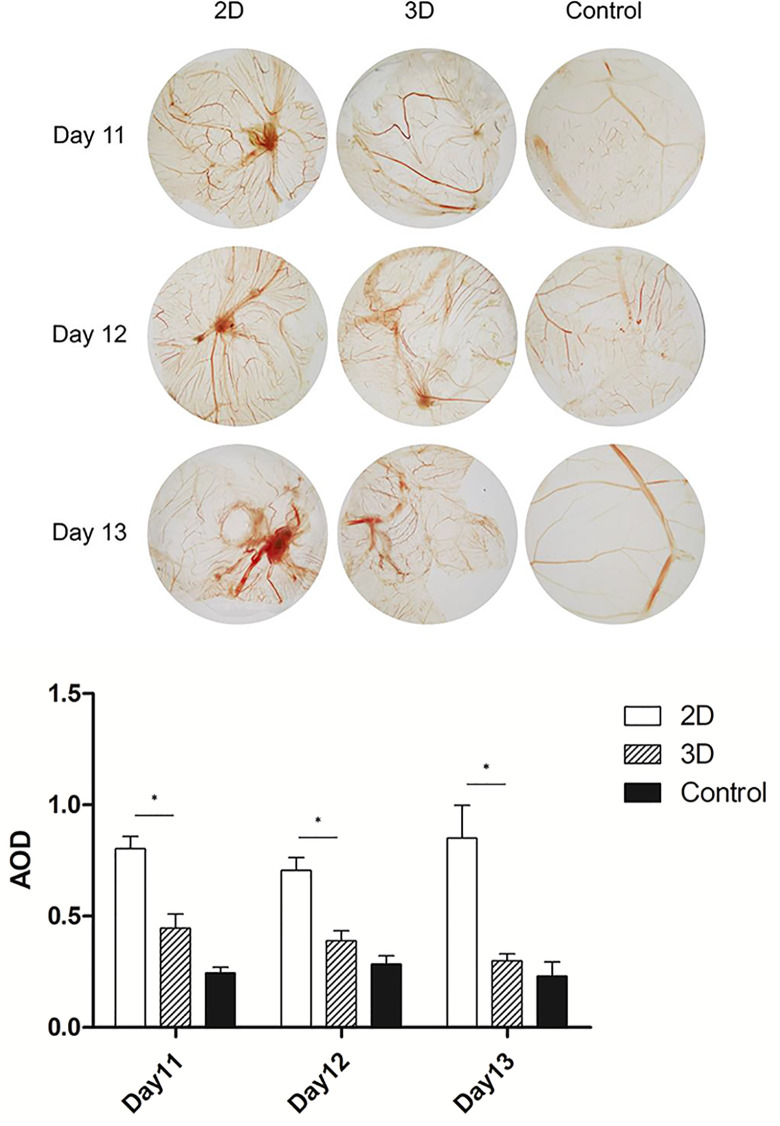
CAM of 11, 12, and 13-day-old chick embryos. Data were obtained from the same area at the same focal length, and were quantified using imageJ software. Error bars represent the SEM (*n* = 3, mean ± SEM; ^*^ *p* < 0.05, Student’s *t*-test).

Pro-angiogenic abilities are an important physiological function of macrophages, so we performed the tubule formation assays and chick embryo chorioallantoic membrane (CAM) assay to detect the effect of 3D culture on these abilities. Tubule formation assays revealed that umbilical vein cells formed sparser and shorter tubules after 3D co-culture with macrophages (**Figure 3**). Moreover, the yolk membrane of chicken embryos stimulated with macrophages cultured for 11, 12, and 13 days had more abundant blood vessels, albeit to different degrees, than the control group (**Figure 4**). When comparing the 3D- and 2D-cultured groups, blood vessels formed from the central stimulus location on the vitelline membrane were more sparse and slender in the 3D-cultured group. In addition, chicken embryo allantoic experiments demonstrated that the pro-angiogenic ability of 3D-cultured macrophages was significantly decreased *in vivo* (**Figure 4**). This experiment only preliminarily showed that 3D-cultured macrophages had reduced ability to promote angiogenesis; the next step was to analyze the molecular mechanisms causing this change.

### 3D-Cultured Macrophages Displayed Different Gene Expression Profiles

**Figure 5:**
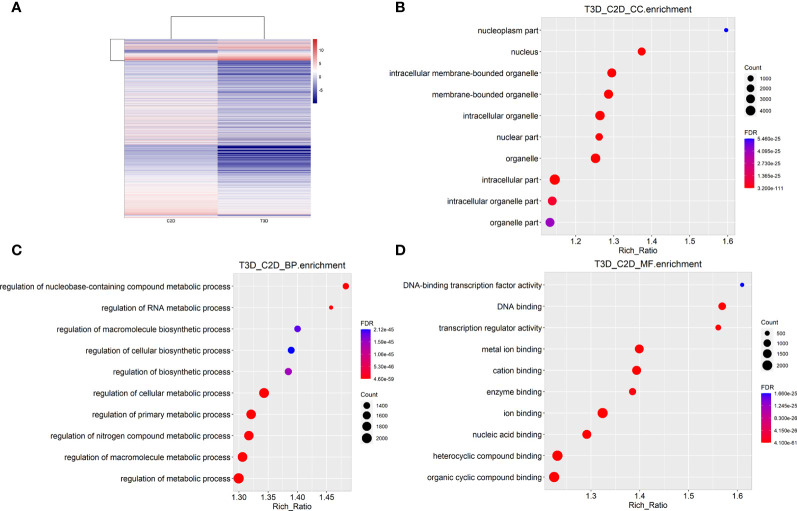
RNA-seq analysis and integration (3D vs. 2D). **(A)** Heat map of gene expression in 2D- and 3D-cultured RAW264.7 cells **(B)** Cellular components enrichment diagram of Q values for single group GO items. **(C)** Biological processes enrichment diagram of Q values for single group GO items. **(D)** Molecular function enrichment diagram of Q values for single group GO items.

Transcriptome sequencing is a common method for rapidly assessing genes with significant changes. The gene expression profiles acquired using RNA-sequencing (RNA-seq) revealed that after 24 h of culture under the same conditions, 3D-cultured macrophages showed significant differences in gene expression. RNA-seq detected 18580 genes in the 2D group and 15777 genes in the 3D group, among which 6762 were differentially expressed (fold change > 2, *p* = 0.05, *p*adj = 0.05) in both males and females. A total of 5949 genes were downregulated and 813 were upregulated. Genes with significantly different expression levels (DEGs; >6 fold) are listed in **Supplementary Table 1**. The five most significant DEGs were Gsta3, Angpt4, Cryab, Cyp2e1, and Ahsg. A gene expression heat map (**Figure 5**) revealed that many genes were upregulated or downregulated to varying degrees in macrophages when cultured using different methods.

**Figure 6:**
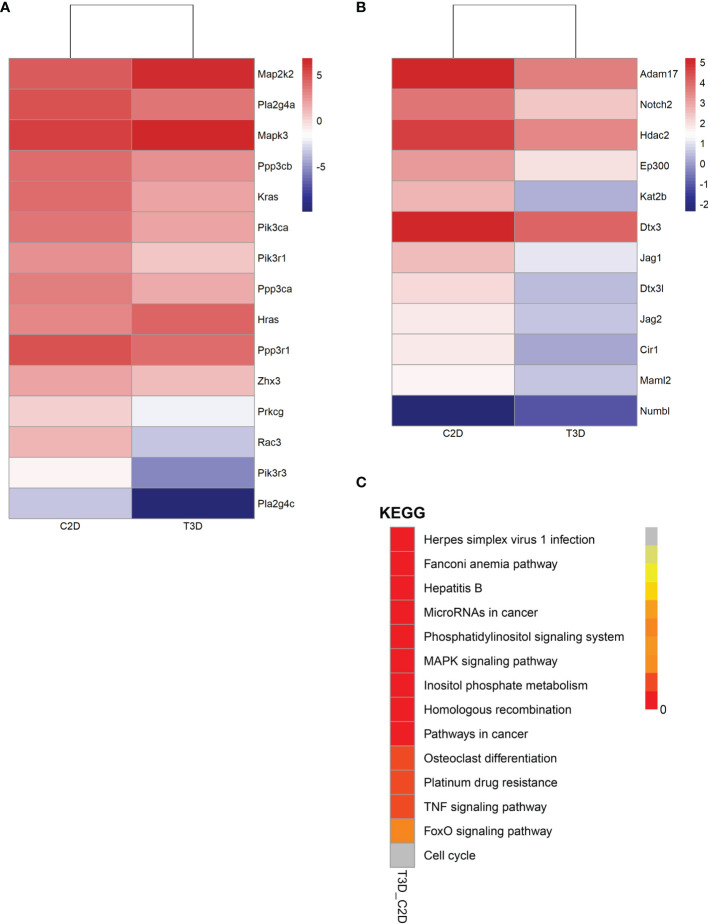
Gene expression of pathways in 2D- and 3D-cultured macrophages (3D vs. 2D). **(A)** Heatmap of gene expression in the VEGF signaling pathway. **(B)** Heatmap of gene expression in the Notch signaling pathway. **(C)** Distribution diagram of Q values for the top fourteen significantly enriched pathways.

**Figure 7:**
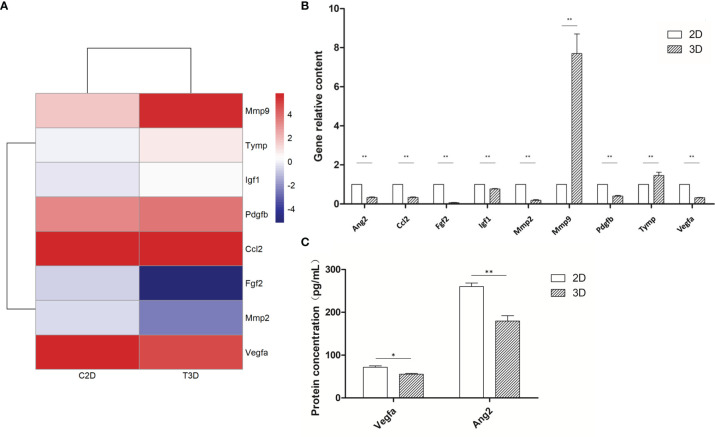
Expression of angiogenesis-related genes of 2D- and 3D-cultured macrophages. **(A)** Heat map of angiogenic-related gene expression in 2D- and 3D-cultured RAW264.7 cells. **(B)** Statistical analysis of the expression of angiogenic factors in 2D- and 3D-cultured macrophages. Data were normalized to mouse β-actin. Ang2, Ccl2, Fgf2, Igf1, Mmp2, Pdgfb, and Vegfa were down-regulated in 3D culture, whereas Mmp9 and Tymp were up-regulated. Error bars represent the SEM (n = 3, mean ± SEM; *p < 0.05, **p < 0.01, Student’s t-test). **(C)** ELISA analysis of Vegfa and Ang2 expression in 2D- and 3D-cultured macrophages. Error bars represent the SEM (n = 3; mean ± SEM; *p < 0.05, **p < 0.01, Student’s t-test).

To provide a comprehensive description of the properties of the DEGs and their gene products, we performed Gene Ontology (GO) analysis (www.geneontology.gov). Five of the most significantly enriched biological processes between the 3D and 2D culture group were the regulation of response to macrophage colony-stimulating factor, t-circle formation, formation of extrachromosomal circular DNA, regulation of cellular response to macrophage colony-stimulating factor stimulus, and elastin metabolic process. The top five significantly enriched cellular components were condensed nuclear chromosome kinetochore, ciliary transition fiber, condensin complex, BBSome, and endoplasmic reticulum tubular network membrane. The top five significantly enriched molecular functions were mRNA methyltransferase activity, 5’-flap endonuclease activity, rRNA (adenine) methyltransferase activity, 5’-3’ exoribonuclease activity, and four-way junction helicase activity (**Figure 5**). KEGG pathway analysis using the KEGG pathway database (www.genome.jp/kegg/pathway.html) identified the top five most significantly enriched pathways between the 3D and 2D culture groups, which were Herpes simplex virus 1 infection, the Fanconi anemia pathway, Hepatitis B, microRNAs in cancer, and phosphatidylinositol signaling (**Figure 6**). We paid special attention to the genes related to angiogenic ability of macrophages, and screened out eight genes that showed significantly different expression: FGF2, MMP2, VEGFA, PDGFB, IGF1, CCL2, MMP9, and TYMP. The expression of PDGFB, IGF1, CCL2, MMP9, and TYMP increased, and the expression of FGF2, MMP2, VEGFA decreased (**Figure 7**). Thus, preliminary RNA-seq screening showed that changing the culture mode caused significant changes in the gene expression of macrophages.

### Two Key Angiogenesis-Related Pathways Were Downregulated in 3D-Cultured Macrophages

Next, we analyzed two vital pathways directly related to angiogenesis: the VEGF signaling pathway and the Notch signaling pathway (**Figure 6** and **Table 1**). After 3D culture, the expression of most genes related to these two pathways was lower than after 2D culture, possibly explaining the decreased angiogenic ability of macrophages in 3D culture.

**Table 1:** Differential gene expression in the VEGF and Notch signaling pathways between 3D- and 2D-cultured macrophages.

Pathway	Gene name	Up/down	Log2 fold change
**VEGF signaling pathway**	Map2k2	up	1.514612
	Pla2g4a	down	-1.51379
	Mapk3	up	1.127462
	Ppp3cb	down	-1.41499
	Kras	down	-2.02601
	Pik3ca	down	-1.53935
	Pik3r1	down	-2.06777
	Ppp3ca	down	-1.41097
	Hras	up	1.463063
	Ppp3r1	down	-1.1316
	Zhx3	down	-1.19361
	Crybg3	down	-1.25967
	Prkcg	down	-2.51374
	Rac3	down	-4.5345
	Pik3r3	down	-4.34185
	Gm12992	down	-2.32805
	Gm13033	down	-2.01993
	Pla2g4c	down	-3.5345
**Notch signaling pathway**	Adam17	down	-1.659438312
	Notch2	down	-1.335282568
	Hdac2	down	-1.275534112
	Ep300	down	-1.066559572
	Kat2b	down	-2.390734442
	Dtx3	down	-1.113109918
	Jag1	down	-1.456806965
	Dtx3l	down	-1.689182933
	Crybg3	down	-1.259669932
	Jag2	down	-1.249199077
	Cir1	down	-1.6518552
	Maml2	down	-1.056699244
	Numbl	up	1.331750362
	A330040F15Rik	down	-1.534498249
	AC132954.5	down	-2.797532655
	Gm42820	down	-4.019925077
	Gm2810	down	-3.019925077
	Gm13803	down	-2.212570155

### 3D-Cultured Macrophages Display Differences in Angiogenesis-Related Gene Expression

RNA-seq analyses revealed that the expression of NADPH, a commonly used internal reference, differed significantly between the 2D and 3D groups; therefore, we used β-actin, which had no significant difference, as the internal reference for subsequent PCR analyses. The mRNA expression of nine macrophage-related angiogenic genes was detected following both 2D and 3D culture. MMP9 and TYMP were significantly upregulated in the 3D culture group, whereas the expression of all other genes was downregulated. The protein expression levels of key angiogenic factors VEGFA and Ang2 were also lower in the 3D-cultured macrophages than in the 2D group, showing the same trend as at the mRNA level. In general, the mRNA expression of most angiogenic factors in 3D-cultured macrophages was decreased, especially that of the two key factors, of which both the mRNA expression and protein expression were significantly down-regulated (**Figure 7**). The decreased expression of these key angiogenic factors may be one of the vital factors causing the decreased angiogenic ability of 3D-cultured macrophages.

## Discussion

The pro-angiogenic capacity of macrophages plays an important role in many pathological and physiological processes. In rheumatoid arthritis, macrophages promote synovitis and bone and cartilage destruction by secreting excessive pro-angiogenic factors that counteract angiogenic inhibitors and support increased trans-endothelial leukocyte infiltration (15, 16). In addition, macrophages exert long-term effects throughout vascular germination and anastomosis in neovascularization during wound repair (17), while macrophage pro-angiogenesis plays an important role in the heart after myocardial infarction (18) and in glioblastoma (19). To more accurately study the effects of 3D culture on macrophages and their angiogenic abilities, we first established a 3D culture system for macrophages. 3D-cultured cells displayed clear changes in cell morphology alongside reduced cell proliferation and an increase in G0 and G1 phase cells, indicating a slower growth rate. These findings are consistent with previous studies, in which the proliferation of glioma cells was reduced in 3D culture, but apoptosis was unaffected, with slow growth attributed to dedifferentiation and reduced proliferation (20). Thus, 3D culture restricts excessively rapid growth, making the genome and transcriptome more stable compared to 2D growth and better reflecting the internal environment of the cell (21).

The results of SEM observation showed that, in contrast to the polygonal flat distribution of cells in 2D culture, cells on 3D scaffolds grew in nearly circular clumps and adhered to the cavity of the scaffold microenvironment. This resulted in non-polar cells, with scattered matrix fibers and variable stiffness. In addition, because cells adhered to the three-dimensional microenvironment, spreading and migration were more difficult, and there was stronger contact between cells, which also increased the transmission of signals between cells. As different cell shapes result in different cytoskeletons, these changes in cell morphology likely led to changes in the physiological function of cells.

To confirm the effect of 3D culture on the angiogenic ability of macrophages *in vivo* and *in vitro*, we conducted tubule formation and CAM assays, respectively. Co-culture and allantoic assays are classic methods for detecting angiogenesis, while CAM is an immunocompetent animal model that can be used for xenotransplantation (22). Since the core amino acid sequence of the angiogenic factors is relatively conservative in this model, it can be used across species (23). Cocultivation and tubule formation assays revealed that the total length of branch nodes in the 3D-cultured group was significantly shorter than in the 2D group, while CAM assays showed a similar trend. These findings are significant because, in addition to cytokines, cells may also secrete exosomes and other small molecules to regulate the microenvironment (24, 25); thus, angiogenic function cannot simply be explained by detecting RNA and protein expression. Together, the results of the tube formation and CAM assays indicate that 3D culture significantly decreased the pro-angiogenesis ability of the cells.

To elucidate the effect of 3D culture on macrophage gene expression, we analyzed the transcriptome using second-generation sequencing technology. Notably, various genes involved in multiple functions were differentially expressed when macrophages were cultured in a 3D system compared to the traditional 2D system. Further analysis revealed significant differences in the expression of various pathways. Angiogenesis is predominantly coordinated by two pathways, namely VEGF and Notch signaling (26). VEGF/VEGFR signaling is a major mediator of blood vessel formation, which induces colorectal proliferation, migration, and survival by activating VEGFR2 and its downstream signal transduction pathways (27). Conversely, the Notch pathway is an intercellular contact-dependent signaling mechanism that is involved in the regulation of cell fate and tissue homeostasis, and also plays important roles in arterial specialization, neovascularization, and vascular maturation. In new blood vessel buds, Notch promotes a distinction between the main “tip” endothelial cells and growing “stem” cells that eventually form new capillaries. Notch signaling also affects vascular stability by regulating the function of vascular wall cells (28–30). Our gene sequencing results indicated that the expression of both pathways was lower in macrophages cultured in 3D than in 2D, and may therefore explain the decreased angiogenic abilities of these cells.

Through transcriptome sequencing, we also identified that eight genes associated with angiogenesis were altered, including FGF2, MMP2, VEGFA, PDGFB, IGF1, CCL2, MMP9, and TYMP. In particular, the gene expression of FGF2, MMP2, and VEGFA decreased, whereas the expression of PDGFB, IGF1, CCL2, MMP9, and TYMP increased. Subsequently, we performed PCR analysis on these eight genes, plus a key angiogenesis-related gene, ANG2. The results showed that the mRNA expression of MMP9 and TYMP increased, and the mRNA expression of ANG2, PDGFB, IGF1, CCL2, FGF2, MMP2, and VEGFA decreased. Furthermore, ELISA assays confirmed decreases in the secretion of various angiogenic factors, particularly VEGFA and Ang2. Thus, one of the key reasons for the decreased angiogenesis ability of macrophages was that the decreased expression of these angiogenesis-related genes led to the decreased secretion of various angiogenic factors.

VEGFA is the most important and potent angiogenesis stimulator that plays important roles in vasculogenesis and neoangiogenesis by promoting cell proliferation, inhibiting apoptosis, increasing vascular permeability and vasodilatation, and recruiting inflammatory cells to the injury site (31–34). Ang2 is a ligand of the endothelial receptor Tie2 that can promote the separation of smooth muscle cells and reduce underlying mechanisms at the sites of angiogenesis and vascular remodeling, thereby easing contact between endothelial cells and allowing their migration (35). In conjunction with VEGF, Ang-2 can also promote neo-vascularization (36); therefore, we selected these two genes for further ELISA detection. The protein expression of both factors was significantly lower in 3D-cultured macrophages, consistent with the findings of both the *in vivo* and *in vitro* experiments. Thus, the decreased angiogenic ability of macrophages after 3D culture could be closely related to the downregulation of VEGF and Ang2 expression.

However, this study had some limitations. The mechanism of 3D culture causing decreased vasogenic ability of macrophages may be multifaceted; for example, changes in exosome miRNA may also need in-depth study. In some cases, angiogenesis is good for body damage repair, but in other cases, angiogenesis may further aggravate illness, such as in diabetic retinopathy angiogenesis. Thus, the next step of work in 3D-cultured macrophage research should be to focus on the specific pathological conditions.

In conclusion, this study demonstrated that macrophages cultured in a 3D environment closer to the microenvironment of biological tissue displayed a lower pro-angiogenic ability than those grown in traditional 2D culture. Moreover, the decreased pro-angiogenic ability of these cells may be due to decreases in the expression of angiogenic factors and important angiogenesis-related pathways caused by 3D culture. These findings are therefore worthy of further investigation and verification, and provide novel avenues for future research in cytology and macrophages.

## Data Availability Statement

The datasets presented in this study can be found in online repositories. The names of the repository/repositories and accession number(s) can be found below: https://www.ncbi.nlm.nih.gov/gds, accession ID: GSE186841.

## Author Contributions

ZL: Funding acquisition, Resources, Project administration. HS: Conceptualization, Writing - review & editing, Formal analysis, Roles/Writing - original draft, Investigation, Data curation. DL: Conceptualization, Methodology, Validation, Writing - review& editing. QS: Resources, Investigation. ZH: Data curation. YT: Investigation, Validation. MQ: Data curation. XM: Data curation. All authors contributed to the article and approved the submitted version.

## Funding

This work was supported by the National Key R&D Program of China [grant number 2018YFC0114707]; the Key R&D Program of Shandong Province [grant number 2018GSF118047]; and the Science and Technology Project of the Jilin Provincial Department of Science and Technology [grant number 20180201029YY].

## Conflict of Interest

The authors declare that the research was conducted in the absence of any commercial or financial relationships that could be construed as a potential conflict of interest.

## Publisher’s Note

All claims expressed in this article are solely those of the authors and do not necessarily represent those of their affiliated organizations, or those of the publisher, the editors and the reviewers. Any product that may be evaluated in this article, or claim that may be made by its manufacturer, is not guaranteed or endorsed by the publisher.
