# Supplementary material for: Three-Dimensional Culture Decreases the Angiogenic Ability of Mouse Macrophages
Source: Front Immunol. 2021 Dec 22;12:795066. doi: 10.3389/fimmu.2021.795066 (PMC8727350; doi:10.3389/fimmu.2021.795066)
Supplement: Supplementary file 1 [file DataSheet_1.docx]

Supplementary Material

# Supplementary Figures and Tables

## Supplementary Figures


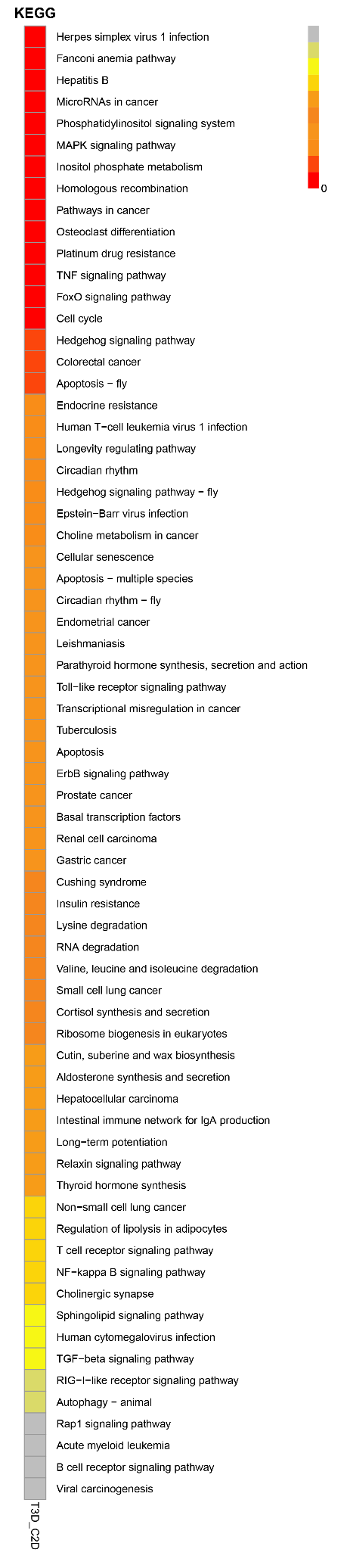


**Supplementary Figure 1.** Distribution diagram of Q values for enriched pathways.

## Supplementary Tables

**Supplementary Table 1.** Nucleotide sequences of primers used for PCR analysis.

| **Name** | **Sequence (5'‑3')** | **Product size, bp** |
| --- | --- | --- |
| β-actin‑F | GGCTGTATTCCCCTCCATCG | 154 |
| β-actin‑R | CCAGTTGGTAACAATGCCATGT |  |
| **Ang2-F** | AAGGAAGCCCTTATGGACGA | 158 |
| **Ang2-R** | GGGGAGACCTTCCTTTGTGTG |  |
| **Fgf2-F** | GCTCTACTGCAAGAACGGCG | 75 |
| **Fgf2-R** | GTTTGACGTGTGGGTCGCT |  |
| **Ccl2-F** | TACAAGAGGATCACCAGCAGC | 95 |
| **Ccl2-R** | CATTCCTTCTTGGGGTCAGCA |  |
| **Igf1-F** | CAGTTCGTGTGTGGACCGAG | 149 |
| **Igf1-R** | AGTGGGGCACAGTACATCTC |  |
| **Mmp2-F** | CAGGGCACCTCCTACAACAG | 127 |
| **Mmp2-R** | CAGTGGACATAGCGGTCTCG |  |
| **Mmp9-F** | TAGATCATTCCAGCGTGCCG | 120 |
| **Mmp9-R** | GCTTAGAGCCACGACCATACA |  |
| **PDGFB-F** | CCCTCGGCCTGTGACTAGAA | 144 |
| **PDGFB-R** | GCCTTGTCATGGGTGTGCTT |  |
| **Tymp-F** | CCGCCATGGATAACCCTCTG | 153 |
| **Tymp-R** | TTTCCGCCTGTCCGCTAATC |  |
| **Vegfa-F** | GCCAGCACATAGGAGAGATGA | 72 |
| **Vegfa-R** | TTGTTCTGTCTTTCTTTGGTCTGC |  |

**Supplementary Table 2.** Differential gene expression between macrophages in 3D and 2D culture.

| **Gene name** | **Fold change (> 6)** | **Description** |
| --- | --- | --- |
| **Gsta3** | 56.9578966 | glutathione S-transferase A3 isoform a [*Mus musculus*] |
| **Angpt4** | 50.05390913 | angiopoietin-4 precursor [*Mus musculus*] |
| **Cryab** | 38.01508099 | PREDICTED: alpha-crystallin B chain [*Pteropus vampyrus*] |
| **Cyp2e1** | 34.51993733 | cytochrome P450 2E1 [*Mus musculus*] |
| **Ahsg** | 32.79394047 | alpha-2-HS-glycoprotein isoform 1 precursor [*Mus musculus*] |
| **Pzp** | 29.34194673 | pregnancy zone protein, isoform CRA_b, partial [*Mus musculus*] |
| **Plg** | 29.34194673 | unnamed protein product [*Mus musculus*] |
| **Gm6576** | 27.61594987 | ribosomal protein S2 [*Rattus norvegicus*] |
| **Alb** | 25.02695457 | serum albumin preproprotein [*Mus musculus*] |
| **Nxph3** | 25.02695457 | neurexophilin-3 precursor [*Mus musculus*] |
| **Apob** | 22.43795927 | apolipoprotein B-100 precursor [*Mus musculus*] |
| **Gm4691** | 22.43795927 | PREDICTED: LOW QUALITY PROTEIN: glyceraldehyde-3-phosphate dehydrogenase [*Colobus angolensis palliatus*] |
| **AC158392.3** | 21.42266699 | enolase 1B, retrotransposed [*Mus musculus*] |
| **Apoa1** | 20.7119624 | apolipoprotein A-I preproprotein [*Mus musculus*] |
| **1190005I06Rik** | 20.7119624 | uncharacterized protein C16orf74 homolog [*Mus musculus*] |
| **Ttr** | 18.98596553 | transthyretin precursor [*Mus musculus*] |
| **Gm11824** | 18.98596553 | mCG2650 [*Mus musculus*] |
| **D830050J10Rik** | 17.25996867 | unnamed protein product [*Mus musculus*] |
| **Phospho1** | 15.5339718 | phosphatase, orphan 1, isoform CRA_a, partial [*Mus musculus*] |
| **Pmepa1** | 15.5339718 | Pmepa1 protein, partial [*Mus musculus*] |
| **Slpi** | 15.5339718 | antileukoproteinase precursor [*Mus musculus*] |
| **Cygb** | 15.5339718 | cytoglobin [*Mus musculus*] |
| **Fetub** | 15.5339718 | PREDICTED: fetuin-B isoform X1 [*Mus musculus*] |
| **Gm17110** | 15.5339718 | . |
| **Gm4575** | 15.5339718 | glyceraldehyde-3-phosphate dehydrogenase isoform 2 [*Mus musculus*] |
| **Mmp9** | 14.19788386 | unnamed protein product [*Mus musculus*] |
| **Cyp4f39** | 13.80797493 | cytochrome P450, family 2, subfamily E, polypeptide 2 homolog [*Mus musculus*] |
| **Gm5921** | 13.80797493 | ribosomal protein S2 [*Rattus norvegicus*] |
| **Gm12164** | 13.80797493 | protein FAM65B [*Rattus norvegicus*] |
| **Rps12-ps1** | 13.80797493 | PREDICTED: 40S ribosomal protein S12 [*Pteropus alecto*] |
| **Gm3534** | 12.9449765 | glyceraldehyde-3-phosphate dehydrogenase isoform 2 [*Mus musculus*] |
| **Tmem179** | 12.08197807 | transmembrane protein 179 [*Mus musculus*] |
| **Dysf** | 12.08197807 | PREDICTED: dysferlin isoform X9 [*Mus musculus*] |
| **Gp1bb** | 12.08197807 | PREDICTED: platelet glycoprotein Ib beta chain [*Microtus ochrogaster*] |
| **Ces3a** | 12.08197807 | Es31 protein, partial [*Mus musculus*] |
| **Gm5356** | 12.08197807 | mCG13639 [*Mus musculus*] |
| **Adh1** | 12.08197807 | alcohol dehydrogenase 1 [*Mus musculus*] |
| **Gm12892** | 12.08197807 | creatine kinase B-type [*Mus musculus*] |
| **Cck** | 11.65047885 | cholecystokinin, isoform CRA_a, partial [*Mus musculus*] |
| **Gm8623** | 11.21897963 | PREDICTED: 40S ribosomal protein S16-like [*Bubalus bubalis*] |
| **Trmt112-ps1** | 11.21897963 | mCG12532 [*Mus musculus*] |
| **Rpl28-ps3** | 10.45751043 | mCG120681 [*Mus musculus*] |
| **Gm9385** | 10.3559812 | PREDICTED: 60S ribosomal protein L24-like [*Bubalus bubalis*] |
| **AC154864.1** | 10.3559812 | LOC72520 protein [*Mus musculus*] |
| **Mlxipl** | 10.3559812 | carbohydrate-responsive element-binding protein [*Mus musculus*] |
| **Pmel** | 10.3559812 | melanocyte protein PMEL precursor [*Mus musculus*] |
| **Cyp1a2** | 10.3559812 | cytochrome P450 1A2 [*Mus musculus*] |
| **Hs3st1** | 10.3559812 | heparan sulfate glucosamine 3-O-sulfotransferase 1 precursor [*Mus musculus*] |
| **9530053A07Rik** | 10.3559812 | Fc fragment of IgG binding protein-like precursor [*Mus musculus*] |
| **Gstp-ps** | 10.3559812 | glutathione S-transferase P 2 [*Mus musculus*] |
| **Gm10290** | 10.3559812 | glyceraldehyde-3-phosphate dehydrogenase isoform 2 [*Mus musculus*] |
| **Gm9619** | 10.3559812 | PREDICTED: glyceraldehyde-3-phosphate dehydrogenase [*Loxodonta africana*] |
| **AC159190.1** | 10.3559812 | SWI/SNF-related matrix-associated actin-dependent regulator of chromatin subfamily E member 1 [*Mus musculus*] |
| **Hist1h2al** | 9.924481983 | PREDICTED: histone H2A.J-like [*Balaenoptera acutorostrata scammoni*] |
| **Mdga1** | 9.492982767 | PREDICTED: MAM domain-containing glycosylphosphatidylinositol anchor protein 1 isoform X1 [*Mus musculus*] |
| **F2rl2** | 9.492982767 | proteinase-activated receptor 3 precursor [*Mus musculus*] |
| **Tmem59l** | 9.492982767 | transmembrane protein 59-like precursor [*Mus musculus*] |
| **Rps15a-ps5** | 9.492982767 | PREDICTED: 40S ribosomal protein S15a isoform X1 [*Gallus gallus*] |
| **Gm12671** | 9.492982767 | glyceraldehyde-3-phosphate dehydrogenase isoform 2 [*Mus musculus*] |
| **Plin2** | 9.268495635 | perilipin-2 [*Mus musculus*] |
| **Fer1l6** | 9.06148355 | PREDICTED: fer-1-like protein 6 isoform X1 [*Mus musculus*] |
| **Ftl1-ps1** | 9.025525282 | ferritin light chain 1 [*Mus musculus*] |
| **Fhod3** | 8.629984333 | FH1/FH2 domain-containing protein 3 isoform 1 [*Mus musculus*] |
| **Fgf18** | 8.629984333 | PREDICTED: fibroblast growth factor 18 [*Fukomys damarensis*] |
| **Gm14928** | 8.629984333 | PREDICTED: ornithine decarboxylase [*Otolemur garnettii*] |
| **Rpl31-ps9** | 8.629984333 | PREDICTED: 60S ribosomal protein L31 [*Pteropus alecto*] |
| **Gm16470** | 8.629984333 | glyceraldehyde-3-phosphate dehydrogenase isoform 2 [*Mus musculus*] |
| **Gm6652** | 8.629984333 | sorbitol dehydrogenase [*Mus musculus*] |
| **Psg18** | 8.629984333 | pregnancy specific glycoprotein 18 isoform 1 [*Mus musculus*] |
| **Krt19** | 8.629984333 | keratin, type I cytoskeletal 19 isoform 1 [*Mus musculus*] |
| **Itih4** | 8.629984333 | inter alpha-trypsin inhibitor, heavy chain 4, isoform CRA_d, partial [*Mus musculus*] |
| **Cdh2** | 8.629984333 | cadherin-2 preproprotein [*Mus musculus*] |
| **Cyp21a1** | 8.629984333 | unnamed protein product [*Mus musculus*] |
| **Papss2** | 8.629984333 | bifunctional 3'-phosphoadenosine 5'-phosphosulfate synthase 2 isoform 1 [*Mus musculus*] |
| **Tekt2** | 8.629984333 | tektin-2 [*Mus musculus*] |
| **2310030G06Rik** | 8.629984333 | uncharacterized protein C11orf52 homolog [*Mus musculus*] |
| **Apoc3** | 8.629984333 | apolipoprotein C-III isoform a [*Mus musculus*] |
| **Ptprf** | 8.629984333 | receptor-type tyrosine-protein phosphatase F precursor [*Mus musculus*] |
| **Galnt9** | 8.629984333 | polypeptide N-acetylgalactosaminyltransferase 9 isoform A [*Mus musculus*] |
| **Nat8f2** | 8.629984333 | probable N-acetyltransferase CML2 [*Mus musculus*] |
| **Fgb** | 8.629984333 | fibrinogen beta chain preproprotein [*Mus musculus*] |
| **Acp7** | 8.629984333 | acid phosphatase type 7 [*Mus musculus*] |
| **Prss45** | 8.629984333 | inactive serine protease 45 precursor [*Mus musculus*] |
| **Gprc5c** | 8.629984333 | G protein-coupled receptor, family C, group 5, member C [*Mus musculus*] |
| **Xcr1** | 8.629984333 | chemokine XC receptor 1 [*Mus musculus*] |
| **Serpina1b** | 8.629984333 | alpha-1-antitrypsin 1-2 precursor [*Mus musculus*] |
| **Gm15922** | 8.629984333 | paired-Ig-like receptor A2 precursor [*Mus musculus*] |
| **Gm13882** | 8.629984333 | unnamed protein product [*Mus musculus*] |
| **Gm3699** | 8.629984333 | . |
| **Gm3436** | 8.629984333 | mCG130637 [*Mus musculus*] |
| **Frmpd2** | 8.629984333 | PREDICTED: FERM and PDZ domain-containing protein 2 isoform X2 [*Mus musculus*] |
| **Hmox1** | 8.4298145 | heme oxygenase (decycling) 1, partial [*Mus musculus*] |
| **Id3** | 7.7669859 | PREDICTED: DNA-binding protein inhibitor ID-3 [*Orcinus orca*] |
| **Gm10359** | 7.7669859 | glyceraldehyde-3-phosphate dehydrogenase isoform 2 [*Mus musculus*] |
| **Gstm2-ps1** | 7.7669859 | mCG131602, isoform CRA_c, partial [*Mus musculus*] |
| **Akr1c12** | 7.7669859 | Aldo-keto reductase family 1, member C12 [*Mus musculus*] |
| **Gm13067** | 7.7669859 | PREDICTED: uncharacterized protein Gm13067 [*Mus musculus*] |
| **Gm6170** | 7.7669859 | PREDICTED: LOW QUALITY PROTEIN: eukaryotic translation elongation factor 1 alpha 1 [*Balaenoptera acutorostrata scammoni*] |
| **Gm37844** | 7.7669859 | mKIAA0711 protein [*Mus musculus*] |
| **Prss46** | 7.683469923 | serine protease 46 [*Mus musculus*] |
| **Gm13464** | 7.479319756 | glyceraldehyde-3-phosphate dehydrogenase isoform 2 [*Mus musculus*] |
| **Ccdc65** | 7.36086899 | coiled-coil domain containing 65, isoform CRA_b, partial [*Mus musculus*] |
| **Gm8399** | 7.335486683 | unnamed protein product [*Mus musculus*] |
| **Ccl22** | 7.335486683 | C-C motif chemokine 22 precursor [*Mus musculus*] |
| **Gm40457** | 7.335486683 | . |
| **Gstp1** | 7.273843938 | glutathione S-transferase P 1 [*Mus musculus*] |
| **Gm10284** | 6.903987467 | glyceraldehyde-3-phosphate dehydrogenase isoform 1 [*Mus musculus*] |
| **Galnt15** | 6.903987467 | UDP-N-acetyl-alpha-D-galactosamine:polypeptide N-acetylgalactosaminyltransferase-like 2, isoform CRA_b, partial [*Mus musculus*] |
| **Rps19-ps4** | 6.903987467 | PREDICTED: 40S ribosomal protein S19 [*Mesocricetus auratus*] |
| **6430573P05Rik** | 6.903987467 | . |
| **Rgcc** | 6.580363054 | PREDICTED: regulator of cell cycle RGCC [*Mesocricetus auratus*] |
| **Gm14760** | 6.558788093 | glyceraldehyde-3-phosphate dehydrogenase isoform 2 [*Mus musculus*] |
| **Gm6204** | 6.505680497 | 40S ribosomal protein S14, partial [*Anas platyrhynchos*] |
| **S100a8** | 6.47248825 | protein S100-A8 [*Mus musculus*] |
| **Gm5507** | 6.47248825 | glyceraldehyde-3-phosphate dehydrogenase isoform 2 [*Mus musculus*] |
| **Rps12-ps24** | 6.47248825 | RecName: Full=40S ribosomal protein S12 |
| **Gm10073** | 6.407109581 | PREDICTED: 60S acidic ribosomal protein P1-like [*Mus musculus*] |
| **Rn7sk** | 6.287560014 | Nuclear prelamin A recognition factor [*Myotis davidii*] |
| **Tmem54** | 6.265144471 | transmembrane protein 54, isoform CRA_c, partial [*Mus musculus*] |
| **Gm3272** | 6.256738642 | glyceraldehyde-3-phosphate dehydrogenase isoform 1 [*Mus musculus*] |
| **Cyp2ab1** | 6.256738642 | hypothetical protein F730023N20, isoform CRA_a [*Mus musculus*] |
| **Rpsa-ps9** | 6.040989033 | protein (40 kD) |
| **Slc9a2** | 6.040989033 | solute carrier family 9 (sodium/hydrogen exchanger), member 2, isoform CRA_a, partial [*Mus musculus*] |
| **Col5a3** | 6.040989033 | collagen alpha-3(V) chain isoform 1 precursor [*Mus musculus*] |
| **Slc28a1** | 6.040989033 | sodium/nucleoside cotransporter 1 [*Mus musculus*] |
| **Areg** | 6.040989033 | amphiregulin preproprotein [*Mus musculus*] |
| **Dennd5b** | 6.040989033 | DENN domain-containing protein 5B [*Mus musculus*] |
| **Prss22** | 6.040989033 | PREDICTED: brain-specific serine protease 4 isoform X1 [*Mus musculus*] |
| **Rplp0-ps1** | 6.040989033 | 60S acidic ribosomal protein P0, partial [*Microtus pennsylvanicus*] |
| **Gm29668** | 6.040989033 | unnamed protein product [*Mus musculus*] |
